# Changing oxidoreduction potential to improve water-soluble yellow pigment production with *Monascus ruber* CGMCC 10910

**DOI:** 10.1186/s12934-017-0828-0

**Published:** 2017-11-21

**Authors:** Tao Huang, Hailing Tan, Fangju Lu, Gong Chen, Zhenqiang Wu

**Affiliations:** School of Biology and Biological Engineering, Guangdong Provincial Key Laboratory of Fermentation and Enzyme Engineering, South China University of Technology, Guangzhou, 510006 China

**Keywords:** Water-soluble *Monascus* yellow pigments, Oxidoreduction potential, NADH/NAD+, Enzyme activity, Pigment biosynthesis genes

## Abstract

**Background:**

*Monascus* pigments are widely used in the food and pharmaceutical industries due to their safety to human health. Our previous study found that glucose concentration induced extracellular oxidoreduction potential (ORP) changes could influence extracellular water-soluble yellow pigment production by *Monascus ruber* CGMCC 10910 in submerged fermentation. In this study, H_2_O_2_ and dithiothreitol (DTT) were used to change the oxidoreduction potential for investigating the effects of oxidative or reductive substances on *Monascus* yellow pigment production by *Monascus ruber* CGMCC 10910.

**Results:**

The extracellular ORP could be controlled by H_2_O_2_ and DTT. Both cell growth and extracellular water-soluble yellow pigment production were enhanced under H_2_O_2_-induced oxidative (HIO) conditions and were inhibited under dithiothreitol-induced reductive conditions. By optimizing the amount of H_2_O_2_ added and the timing of the addition, the yield of extracellular water-soluble yellow pigments significantly increased and reached a maximum of 209 AU, when 10 mM H_2_O_2_ was added on the 3rd day of fermentation with *M. ruber* CGMCC 10910. Under HIO conditions, the ratio of NADH/NAD+ was much lower than that in the control group, and the expression levels of relative pigment biosynthesis genes were up-regulated; moreover, the activity of glucose-6-phosphate dehydrogenase (G6PDH) was increased while 6-phosphofructokinase (PFK) activity was inhibited.

**Conclusions:**

Oxidative conditions induced by H_2_O_2_ increased water-soluble yellow pigment accumulation via up-regulation of the expression levels of relative genes and by increasing the precursors of pigment biosynthesis through redirection of metabolic flux. In contrast, reductive conditions induced by dithiothreitol inhibited yellow pigment accumulation. This experiment provides a potential strategy for improving the production of *Monascus* yellow pigments.

**Electronic supplementary material:**

The online version of this article (10.1186/s12934-017-0828-0) contains supplementary material, which is available to authorized users.

## Background

*Monascus* pigments are mixed pigments with three main colours: yellow, orange and red [1]. Recently, *Monascus* yellow pigments have been widely researched due to their anti-tumour [2, 3], anti-inflammation [4], anti-obesity [5], anti-diabetic, and anti-oxidative stress capabilities [6], and their ability to improving memory and learning ability [7], counteract metabolic syndromes [8], and treat liver fibrosis [9]. A large body of evidence indicates that *Monascus* yellow pigments are beneficial to human health in appropriate doses and can potentially be used as nutraceutical or therapeutic agents.

In submerged fermentation, *Monascus* yellow pigments generally consist of a large amount of intracellular alcohol-soluble pigments and a small amount of extracellular water-soluble pigments [10]. Water-soluble yellow pigments have been a popular area of research due to their high stability and convenient utilization [11]. Water-soluble yellow pigments can be synthesized via chemical modification of intracellular alcohol-soluble pigments [12], but this strategy is a potential risk in food applications. In recent decades, considerable effort has been focused on enhancing water-soluble yellow pigment production, such as strain screening [13, 14], strain mutation [15], gene modification [16], and medium optimization [17, 18]. Chen has shown that both extracellular and intracellular yellow pigments are the main pigments produced during long periods of high cell density culturing by *Monascus anka* [19]. In addition, our previous studies found that extracellular water-soluble yellow pigments can be produced by *M. ruber* CGMCC 10910 using high glucose concentrations with low oxidoreduction potential (ORP) [20, 21]. However, the pigment yield and glucose utilization was still low.

Oxidoreduction potential was identified as a control parameter for fermentation processes [22–24]. Intracellular ORP is primarily determined by the ratio of NADH/NAD+ [25]. High NADH/NAD+ ratios can regulate intracellular metabolites to generate unusable byproducts, such as alcohol and lactate [26]. However, intracellular ORP can be influenced by changing extracellular ORP [24], while extracellular ORP can be changed by adding oxidative or reductive substances, such as H_2_O_2_, dithiothreitol (DTT), and potassium ferricyanide [24, 27]. It has been reported that spinosad and pseudoaglycone yields increased 3.11-fold in *Saccharopolyspora spinosa* fermentation under HIO conditions, and high citric acid productivity could be achieved in *Aspergillus niger* cultures with defined ORP profiles [22, 28]. Therefore, extracellular ORP provides an alternative parameter for the optimization of metabolic production. *Monascus* pigment biosynthesis occurs via a polyketide pathway and requires many primary metabolites, such as acetyl-CoA, malonyl-CoA, and NADPH [1, 31, 32]. The pentose phosphate pathway (PPP), a branch pathway of glycometabolism, was shown to be the primary source of NADPH for polyketide and lipid biosynthesis [29]. Recently, the biosynthetic gene cluster of *Monascus* pigments in the *M. purpureus* and *M. ruber* genome and the functions of some critical genes involved in the pigment biosynthetic pathway were reported [30, 31]. Both the targeted inactivation of *MpPKS5* (the homolog of *MpigA* in *M. ruber*) in *M. purpureus* and the targeted-deletion of *MpigA* in *M. ruber* resulted in the abolishment of pigment production, confirming that polyketide synthase is involved in pigment biosynthesis [31, 32]. The genes *MpFasA2* (the homolog of *MpigJ* in *M. ruber*) and *MpfasB2* (the homolog of *MpigK* in *M. ruber*) respectively encode fatty acid synthase α-subunit and fatty acid synthase β-subunit and supply the medium-chain (C8 and C10) fatty acyl moieties for *Monascus* pigments biosynthetic [31–34]. The product profile of *mppA* (the homolog of *MpigC* in *M. ruber*), *mppC* (the homolog of *MpigE* in *M. ruber*) and *mppE* (the homolog of *MpigG* in *M. ruber*) mutants of *M. purpureus* substantiate that MppA-mediated ω-2 ketoreduction is a prerequisite for the synthesis of the pyranoquinone bicyclic core of the *Monascus* pigments and MppC activity determines the regioselectivity of the spontaneous Knoevenagel condensation. The *mppB* (the homolog of *MpigD* in *M. ruber*) in *M. purpureus* gene encodes a trichothecene 3-*O*-acetyltransferase (AT), which can transfer the medium-chain (C8 and C10) fatty acyl group into the polyketide chromophore to complete pigment biosynthesis. The *mppD* (the homolog of *MpigF* in *M. ruber*) gene in *M. purpureus* encodes an amine oxidase/esterase. The *mppR1* (the homolog of *MpigB* in *M. ruber*) and *mppR2* (the homolog of *MpigI* in *M. ruber*) genes in *M. purpureus* are regulatory genes encode transcription factors for pigments biosynthesis [30, 31, 34–36]. Those reports provide a frame of reference from which to investigate external factors on pigment production at the molecular level.

In this study, H_2_O_2_ was added to the fermentation medium of *M. ruber* CGMCC 10910. Its concentration and addition time were also optimized to improve extracellular water-soluble yellow pigment production. In addition, the effect of the oxidative environment induced by H_2_O_2_ on the extracellular and intracellular ORP, the expression levels of pigment biosynthesis genes, and the activities of key enzymes (PFK and G6PDH) in the glycolysis and pentose phosphate pathway (PPP) were also investigated.

## Methods

### Microorganism

*Monascus ruber* CGMCC 10910, which was deposited at the China General Microbiological Culture Collection Center (CGMCC), was used in this study, and it was cultivated on a potato dextrose agar (PDA) medium at 30 °C for 7 days and then stored at 4 °C.

### Fermentation conditions

The seed culture medium contained 2% glucose, 0.3% yeast extract, 1% peptone, 0.4% KH_2_PO_4_, 0.05% KCl, and 0.001% FeSO_4_·7H_2_O. The fermentation culture medium contained 15% glucose, 0.5% (NH_4_)_2_SO_4_, 0.5% KH_2_PO_4_, 0.05% MgSO_4_·7H_2_O, 0.05% KCl, 0.003% MnSO_4_·H_2_O, 0.001% ZnSO_4_·7H_2_O, and 0.001% FeSO_4_·7H_2_O. For the seed culture, 5–6 loopfuls of single colonies (approximately 10 mm diameter) were scraped off an agar plate and inoculated into a 250 mL Erlenmeyer flask containing 50 mL of seed culture medium and agitated at 180 rpm at 30 °C. After 25 h, 2 mL of seed culture was used to inoculate a 250 mL Erlenmeyer flask containing 25 mL of fermentation culture medium, which was then fermented for 12 days with an agitation speed of 180 rpm at 30 °C. Three duplicates were carried out for each condition.

### Addition method of H_2_O_2_ and dithiothreitol (DTT)

H_2_O_2_ and DTT were filter-sterilized before being added to the medium. One, 5, 10, 15 or 20 mM H_2_O_2_ was added to the medium on day 2 of fermentation to study the effect of H_2_O_2_ concentration on cell growth and yellow pigment production. Ten millimolar H_2_O_2_ was added to the medium on day 0, 1, 3, and 5 of fermentation to study the effect of H_2_O_2_ addition time on cell growth and yellow pigment production. Three gram per liter DTT was added to the medium on day 3 to study the extracellular reducing conditions on cell growth and yellow pigment production.

### Determination of glucose concentration, extracellular oxidoreduction potential (ORP), and DCW

After being cultured, the fermentation broth was vacuum-filtered through a 0.8 mm mixed cellulose esters membrane. The filtrate (extracellular broth) was appropriately diluted to determine the residual glucose concentration using the standard 3,5-dinitrosalicylic acid (DNS) method. Extracellular ORP was detected via an oxidation–reduction electrode (Leici, Shanghai) [20]. After filtration, the mycelia were washed 3 times with distilled water and dried at 60 °C in an oven for 12 h, until a constant mycelia weight was reached to determinate dry cell weight (DCW) by gravity.

### Pigment analysis using UV–visible spectrophotometer

The filtrate (extracellular broth) was appropriately diluted to determine the extracellular water-soluble yellow pigment concentration. The absorbance spectrum of the water-soluble yellow pigments was recorded by a UV–visible spectrophotometer (Unico, USA) from 300 to 550 nm at 1 nm intervals, and the absorbance units (AU) at the peak wavelength of 350 nm was multiplied by the dilution ratio, which was used as an index for the water-soluble yellow pigment concentration [20].

The intracellular pigment concentration was determined according to the following the procedure: the harvested and washed mycelia were resuspended in 25 mL acidic aqueous ethanol (70% v/v, pH = 2 with hydrochloric acid) and incubated for 1 h. The suspension was filtered with filter paper, and the filtrate (intracellular extract) was appropriately diluted to determine intracellular pigment concentration. The absorbance spectrum of the intracellular pigments was recorded by a UV–visible spectrophotometer (Unico, USA) from 300 to 550 nm at 1 nm intervals, and the AU at the peak wavelength of 410 nm were multiplied by the dilution ratio, which was used as an index for the intracellular yellow pigment concentration.

### Analyses of pigment compositions by HPLC

Analyses of pigment compositions were performed using an Alliance e2695 HPLC system (Waters, Milford, CT, USA) equipped with a 2998 Photodiode Array (PDA) detector (Waters, Milford, CT, USA) and a Zorbax Eclipse Plus C18 column (250 × 4.6 mm, 5 μm, Agilent, Palo Alto, CA, USA). The temperature of the column oven was set at 30 °C. A mixture of H_3_PO_4_ solution (pH 2.5, phase A) and acetonitrile (phase B) were used as the mobile phase using the following gradient program: 0 min, 80% A, 20% B; 25 min, 20% A, 80% B; 35 min, 20% A, 80% B; 36 min, 80% A, 20% B; 41 min, 80% A, 20% B. The PDA was set at 200–600 nm, and the flow rate of the mobile phase was 0.8 mL/min [21].

### Determination of the pigment composition using LC–MS

LC–MS (liquid chromatography–mass spectrometry) consisted of a HP1100 HPLC system (Agilent, Palo Alto, CA, USA) and a microTOF-QII mass spectrometer (Bruker, Rheinstetten, Germany). The C18 column and chromatographic conditions were the same as mentioned above, except for mobile phase A (water, 0.1% formic acid).

### NADH and NAD+ determination

The intracellular NADH and NAD+ concentrations were determined using procedures described in previous studies [25, 37]. Mycelia were rapidly ground into a powder using liquid nitrogen; 0.1 g of mycelia powder was quickly transferred into a sample tube containing 1 mL of acidic extracting solution (0.2 M HCl for NAD+) or alkaline extracting solution (0.4 M KOH for NADH). Next, the samples were heated at 50 °C for 10 min and then centrifuged at 12,000*g*, 4 °C for 10 min. The supernatant transformed into a tube and was neutralized using alkaline extracting solution or acidic extracting solution. After neutralization, the samples were centrifuged at 12,000*g*, 4 °C for 10 min. The supernatant was collected for NAD+ and NADH determination using the Coenzyme I NAD(H) content test kit (Nanjing Jiancheng Bioengineering Institute, Nanjing, China) according to the manufacturer’s instructions. The kit is based on an enzymatic cycling assay method.

### Determination of PFK and G6PDH enzyme activities

Mycelia were rapidly ground into a powder using liquid nitrogen; 0.1 g of mycelia powder was quickly transferred into a sample tube containing 1 mL of crude enzyme extracting solution (25 mM Tris–HCl (pH = 7.6), 10 mM MgCl_2_, 20 mM NH_4_Cl, 0.5 mM DTT) and then fully shocked [38, 39]. The protein concentration was quantified using the standard Bradford method. Activity of 6-phosphofructokinase (PFK) was determined using a PFK test kit (Nanjing Jiancheng Bioengineering Institute, Nanjing, China) according to the manufacturer’s instructions. Activity of glucose-6-phosphate dehydrogenase (G6PDH) was analysed as reported with minor modifications [39]. A total of 3 mL of the reaction mixture containing 600 µL of 0.2 M Tris–HCl (pH = 8.0), 150 µL of 5 mM glucose-6-phosphate, 50 µL of 5 mM NADP+, 2 µL of β-mercaptoethanol, and 20 µL of crude enzyme solution was incubated at 30 °C for 20 min. The G6PDH activity of each sample was then determined spectrophotometrically by measuring the formation of NADPH at 340 nm.

### Gene expression analysis

The relative expression levels of key genes involved in pigment biosynthesis were analysed using real-time quantitative PCR as described previously [21]. Mycelia were collected for total RNA extraction using the Plant RNA Extraction Kit (TaKaRa MiniBEST). First, cDNA was synthesized using the PrimeScript™ RT reagent Kit with gDNA Eraser (TaKaRa) according to the supplier’s protocol. Primers for the amplification of *MpFasA2*, *MpFasB2*, *MpPKS5*, *mppR1*, *mppA, mppB*, *mppC*, *mppD*, *mppE*, *mppR2* (GenBank Accession No. KC148521) and the actin gene (GenBank Accession No. AJ417880) are listed in Additional file 1: Table S1. The actin gene was used as a reference gene. Gene expression was monitored by RT-qPCR using SYBR Premix Ex TaqII (TaKaRa). RT-qPCR was performed using a LightCycler 96 (Roche, USA) with the following cycling program: pre-incubation at 95 °C for 30 s, followed by a two-step amplification (40 cycles of denaturation at 95 °C for 5 s, and annealing at 60 °C for 30 s) and dissociation curve analyses (at 95 °C for 10 s, annealing at 65 °C for 60 s, then collection of dissociation curves from 65 to 95 °C, with a final incubation at 97 °C for 1 s).

### Statistical analysis

Each experiment was repeated in triplicate, at minimum. Numerical data are presented as the mean ± SD. The differences among the various treatments were analysed using one-way ANOVA. All statistical analyses were performed by using SPSS 22.0 software, and *p* < 0.05 and *p* < 0.01 were considered significant and highly significant, respectively.

## Results

### Water-soluble yellow pigment production from different H_2_O_2_ concentrations and adding times

**Fig. 1:**
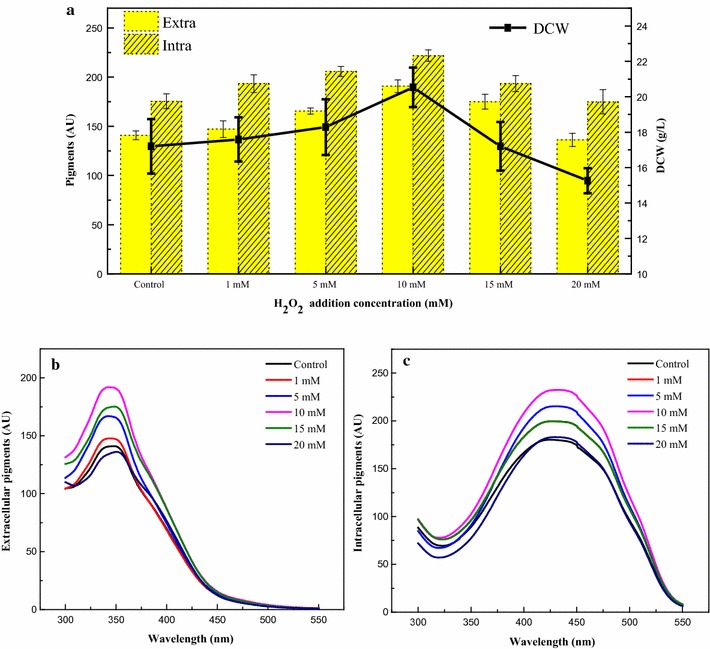
Pigment production by adding different concentrations of H_2_O_2_ on day 2. **a** DCW and yield of extracellular and intracellular yellow pigments. **b** Spectra of extracellular pigments. **c** Spectra of intracellular pigments

*Monascus ruber* CGMCC 10910 has the potential to produce extracellular water-soluble yellow pigments with a maximum absorbance wavelength of 350 nm [20], and the extracellular water-soluble yellow pigments could reach a maximum yield of 147 AU_350_ under high glucose stress fermentation [21]. Further analysis was shown that the extracellular water-soluble yellow pigments mainly contained four kinds of yellow pigments (Additional file 2: Figure S1). The cell growth and pigment production achieved by adding various amounts of H_2_O_2_ to the medium on the 2nd day of *M. ruber* CGMCC 10910 fermentation under high glucose stress are shown in Fig. 1. The results showed that lower amounts of H_2_O_2_ could promote cell growth while higher amounts of H_2_O_2_ had an inhibitory effect on cell growth (Fig. 1a). Extracellular and intracellular pigments were enhanced under certain doses of H_2_O_2_ but were inhibited by high concentrations (Fig. 1a–c). The optimal amount of H_2_O_2_ was found to be 10 mM, which had a 19% increase in dry cell weight (DCW), and a 35 and 26% increase in extracellular and intracellular yellow pigment yield, respectively. Thus, 10 mM was chosen as the suitable concentration for the addition time experiments.

**Fig. 2:**
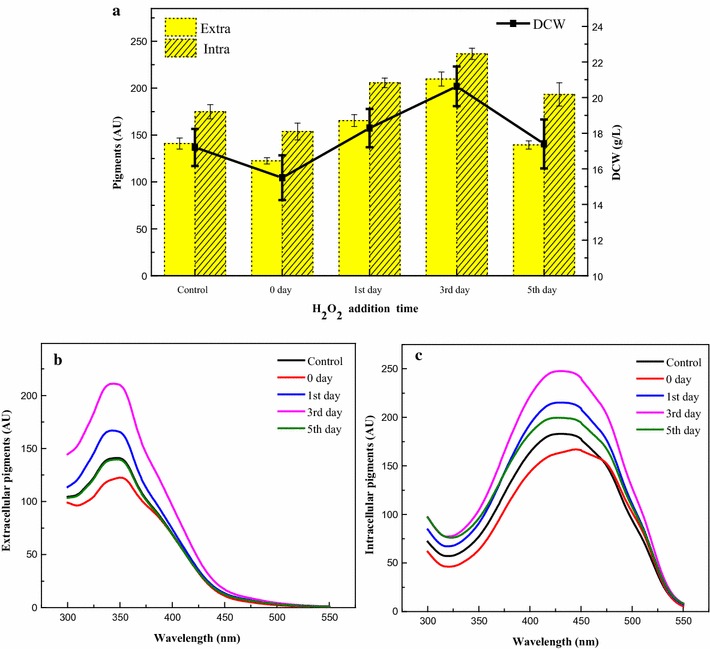
Pigment production by adding 10 mM H_2_O_2_ at different fermentation times. **a** DCW and yield of extracellular and intracellular yellow pigments. **b** Spectra of extracellular pigments. **c** Spectra of intracellular pigments

The optimized concentration, 10 mM H_2_O_2_, was added to the fermentation medium at various growth time points, including at day 0 (beginning of fermentation), the 1st day (early exponential phase), the 3rd day (middle exponential phase), and the 5th day (late exponential phase) during pigment fermentation. Cell growth and pigment production, including intracellular and extracellular yellow pigments, were inhibited when H_2_O_2_ was added at the beginning of fermentation (Fig. 2), and these parameters were marginally increased when H_2_O_2_ was added at the late exponential phase. However, when H_2_O_2_ was added at the middle exponential phase, cell growth and pigment production were much improved. The optimal H_2_O_2_ addition time was on the 3rd day (middle exponential phase), in which extracellular water-soluble yellow pigment yield reached approximately 209 AU_350_, 42% higher than that of the control (Fig. 2a, b), and intracellular yellow pigments also increased by 35% and reached a maximum of approximately 236 AU_410_ (Fig. 2c). The UV–visible spectra and mass spectra of intracellular yellow pigments were compared with literature data (Additional file 3: Figure S2) [40, 41], which were identified as the two well-known pigments, monascin and ankaflavin. Monascin was the mainly intracellular yellow pigment based on the HPLC profile (Additional file 4: Figure S3), and the maximum yield of monascin under HIO conditions was 467.75 μg/mL.

### Water-soluble yellow pigment production under HIO conditions and DTT-induced reductive conditions

**Fig. 3:**
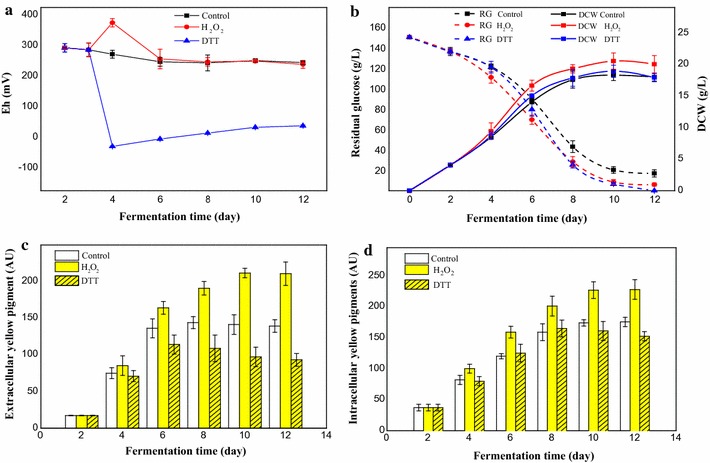
Pigment production time curve after adding H_2_O_2_ or DTT. **a** Extracellular ORP. **b** Glucose consumption and DCW. RG is the abbreviation for residual glucose. **c** Extracellular yellow pigments. **d** Intracellular yellow pigments

H_2_O_2_ acting as an electron acceptor could cause an extracellular oxidative condition (higher extracellular ORP), while dithiothreitol (DTT) acting as a reducing agent could reduce the extracellular ORP [24]. 10 mM of H_2_O_2_ was added to the medium on the 3rd day to create an extracellular oxidative environment, and 3 g/L DTT was added on the 3rd day to create an extracellular reducing environment. Extracellular ORP increased immediately after H_2_O_2_ addition and reached its highest level on day 4, then decreased to a low level, similar to the control, in the later phase of the fermentation (Fig. 3a). A low extracellular ORP was also promptly obtained when DTT was added to the medium. The cell growth rate increased under an oxidative environment, which resulted in a 19% increase in dry cell weight (DCW), while the DCW under a reducing environment increased only slightly by less than 5% (Fig. 3b). The glucose consumption rate increased immediately upon addition of H_2_O_2_, and the total glucose consumption was higher than that of the control. However, the glucose consumption rate under reducing conditions increased only on day 6 and resulted in the lowest residual glucose concentration (Fig. 3b).

The extracellular water-soluble yellow pigment reaching maximum productivity on day 10 under oxidative conditions and reached 209 AU_350_, which was 42% higher than that of the control. However, the maximum productivity of the control was reached on day 8. In contrast, the yield of extracellular water-soluble yellow pigments under reducing conditions was significantly decreased (Fig. 3c). Under oxidative conditions, the yield of extracellular water-soluble yellow pigments based on glucose consumption corresponded to approximately 1.45 AU/g, which was 37.6% higher than that of the control. The generation rate of intracellular yellow pigments was increased under oxidative conditions (Fig. 3d). The yield of intracellular yellow pigments under oxidative conditions based on glucose consumption corresponded to approximately 1.54 AU/g, which was 26.4% higher than that of the control. In contrast, the yield of intracellular yellow pigments under reducing conditions decreased in the later stages of fermentation and resulted in a lower overall yield (Fig. 3d). The yields of extracellular and intracellular yellow pigments per unit DCW under oxidative conditions were higher than that of the control, while those under reducing conditions were lower than the control (Table 1). Which demonstrated that the oxidative conditions improved yellow pigments productivity, while reducing condition played an opposite role. Therefore, extracellular oxidative conditions were better for yellow pigment production.

**Table 1:** Pigments yield per g DCW under HIO conditions and DTT-induced reductive conditions

Fermentation time (day)	Extracellular yield (AU/g DCW)^a^			Intracellular yield (AU/g DCW)^b^		
	Control	H_2_O_2_	DTT	Control	H_2_O_2_	DTT
0	–	–	–	–	–	–
2	4.21 ± 0.05	4.21 ± 0.05	4.21 ± 0.05	9.21 ± 0.16	9.21 ± 0.16	9.21 ± 0.16
4	8.77 ± 0.15	9.03 ± 0.12a	8.11 ± 0.24b	9.63 ± 0.21	9.64 ± 0.09	9.18 ± 0.31a
6	8.67 ± 0.09	9.83 ± 0.29a	7.56 ± 0.25b	8.56 ± 0.16	9.55 ± 0.08a	8.32 ± 0.29
8	9.17 ± 0.34	9.86 ± 0.16a	6.08 ± 0.28b	9.04 ± 0.27	10.41 ± 0.23a	9.23 ± 0.41
10	7.69 ± 0.16	10.47 ± 0.12a	5.11 ± 0.34b	9.50 ± 0.41	11.04 ± 0.21a	8.51 ± 0.43b
12	7.70 ± 0.04	10.24 ± 0.08a	5.18 ± 0.45b	9.04 ± 0.25	11.36 ± 0.40a	8.50 ± 0.18b

The values are expressed are means ± standard deviations, n = 3 for each treatment

Different letters in row represent statistically different mean values (p < 0.05) compared with control

^a^Extracellular yellow pigments yield per g DCW

^b^Intracellular yellow pigments yield per g DCW

### Intracellular NADH/NAD+ levels under HIO conditions

**Fig. 4:**
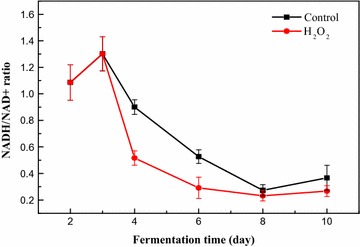
NADH/NAD+ ratio under HIO condition

As shown in Fig. 4, the ratios of NADH/NAD+ under HIO and control conditions were increased and reached 1.3 on the 3rd day of fermentation. From the 3rd day to the 6th day, a large amount of energy was needed to transport intracellular nutrients to support the fast growth of mycelia and to biosynthesize metabolic intermediates. Consequently, the formation rate of NADH was less than its consumption rate during the growth stage, which resulted in a sharp decrease of the NADH/NAD+ ratio during this stage. The decrease rate of the NADH/NAD+ ratio under HIO conditions was higher than that of the control and resulted in a lower NADH/NAD+ ratio in the later exponential phase and stationary phase of the fermentation process. These results indicate that the intracellular oxidoreduction status in *M. ruber* CGMCC 10910 was significantly influenced by H_2_O_2_ addition.

### Expression of pigment biosynthesis genes under HIO conditions

**Fig. 5:**
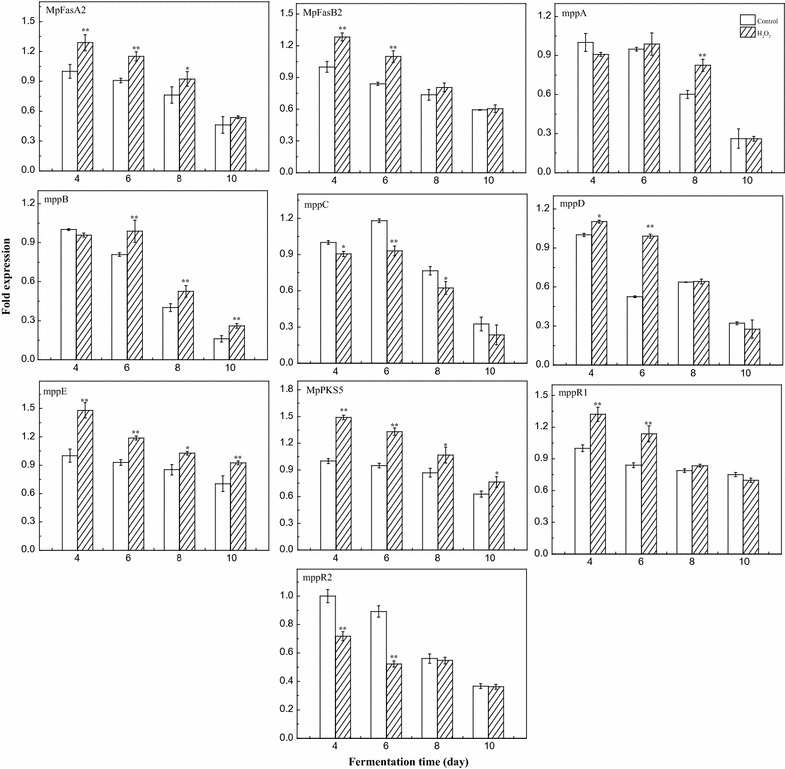
Relative expression levels of the pigment biosynthesis-related genes *MpFasA2*, *MpFasB2*, *MpPKS5*, *mppA*, *mppB*, *mppC*, *mppD*, *mppE*, *mppR1* and *mppR2* under HIO conditions. Error bars represent the standard deviation (n = 3). **p < 0.01, *p < 0.05

The pigment biosynthetic gene cluster has been identified in *Monascus* spp. [31]. The expression of relative pigment biosynthetic genes under HIO conditions was analysed (Fig. 5). The *MpPKS5* gene encodes a pigment polyketide synthase, which is responsible for pigment biosynthesis. *MpfasA2* and *MpfasB2* encode a fungal fatty acid synthase, which produces medium-chain (C8 and C10) fatty acyl moieties, and then a trichothecene 3-*O*-acetyltransferase (AT), encoded by the *mppB* gene, transfers the medium-chain (C8 and C10) fatty acyl groups onto the polyketide chromophore to complete pigment biosynthesis. *mppA, mppC,* and *mppE* encode oxidoreductases, of which a reductive enzyme encoded by *mppE* controls the biosynthesis of yellow pigments. The gene *mppD* encodes an amine oxidase/esterase. The genes *mppR1* and *mppR2* are regulatory genes for pigment biosynthesis [31, 32].

The expression levels of the genes *MpFasA2*, *MpFasB2*, *MpPKS5*, *mppA*, *mppB*, *mppD*, *mppE*, and *mppR1* were significantly up-regulated (*p* < 0.01 or *p* < 0.05) under HIO conditions (Fig. 5). This corroborates findings regarding the intracellular and extracellular pigment production under oxidative conditions (Figs. 1, 2), indicating that H_2_O_2_ stimulates pigment production via up-regulation of transcript levels of these genes. However, the genes *mppC* and *mppR2* were significantly down-regulated (*p* < 0.01 or *p* < 0.05) and were negatively correlated with pigment production. It has been reported that *mppR2* is a negative regulatory factor [42].

### Variation of PFK and G6PDH activities under HIO conditions

**Fig. 6:**
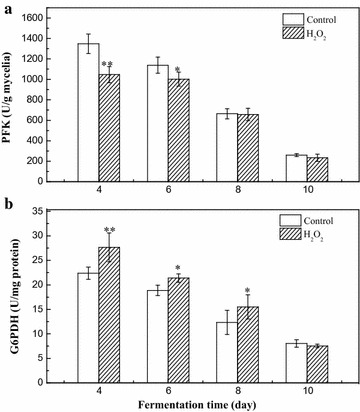
Activities of PFK and G6PDH under HIO conditions. **a** PFK activity. **b** G6PDH activity. *p < 0.05, **p < 0.01

To understand the physiological consequences of *M. ruber* CGMCC 10910 caused by HIO conditions, the activities of key redox-dependent enzymes (PFK and G6PDH) in glycolysis and the pentose phosphate pathway (PPP) were analysed. As shown in Fig. 6a, PFK activity was inhibited between the 4th day and the 6th day under HIO conditions, which indicated that the glycolysis pathway was weakened when extracellular oxidative conditions were induced by H_2_O_2_. At the same time, G6PDH activity was enhanced between the 4th day and the 8th day under HIO conditions, which also indicated that the PPP was enhanced under HIO conditions (Fig. 6b). With the metabolic activities weakening, the PFK activity under HIO conditions became equal to that of the control on the 8th day, while G6PDH activity on the 8th day was also higher than that of the control and achieved levels similar to those of the control on the 10th day.

The responses of PFK and G6PDH activities to HIO conditions in this study suggest that there was a metabolic flux redirection from glycolysis to the PPP in *M. ruber* CGMCC 10910 fermentation, similar to that reported by other studies [28, 38, 39]. The higher G6PDH activity under HIO conditions suggested a higher PPP metabolic reaction rate, thus providing the pigment biosynthesis pathway with ample precursors.

## Discussion

*Monascus* pigments are mixtures with multiple components [1, 10, 43]. It is difficult to determine the concentration of *Monascus* pigments with standard HPLC methods. Thus, the concentration of *Monascus* yellow pigments in this study was represented by the absorbance at their characteristic wavelengths (350 and 410 nm) [20, 21]. In this study, H_2_O_2_ was added at different fermentation time points to study the effects of H_2_O_2_ addition time on cell growth and yellow pigment production in *M. ruber* CGMCC 10910. Cell growth and yellow pigment production were both inhibited when H_2_O_2_ was added at the beginning of fermentation (Fig. 2), indicating the toxic effect of H_2_O_2_ on the conidia germination of *M. ruber* [44]. Cell growth and metabolic activities were slower at the late exponential phase; thus, there was not a significant effect on cell growth and yellow pigment production when H_2_O_2_ was added at this phase. However, extracellular water-soluble yellow pigment production was enhanced by more than 42%, and the DCW increased when H_2_O_2_ was added at the middle exponential phase (Fig. 2), a phase in which cell growth and metabolic activity were robust. The optimal amount of H_2_O_2_ for yellow pigment production was 10 mM; lower amounts of H_2_O_2_ were not sufficient to enhance yellow pigment production, while higher amounts of H_2_O_2_ had an inhibitory effect on cell growth and pigment production (Fig. 1). This kind of dose-dependent induction was also observed in other fermentation systems such as *Taxus chinensis* and *Streptomyces hygroscopicus 5008* [39, 45]. Because the addition of H_2_O_2_ can improve the yield of yellow pigments, additional investigations using the *M. ruber* CGMCC10910 fermentation system were performed.

H_2_O_2_ is an electron acceptor, which can induce an extracellular oxidative environment (higher extracellular ORP) when added to the fermentation medium [24]. Meanwhile, DTT acts as a reducing agent and can decrease extracellular ORP, thereby creating reducing conditions in the extracellular environment [46]. In this study, H_2_O_2_ and DTT were used to modify the extracellular ORP to study the effect of extracellular ORP changes on the water-soluble yellow pigment production in *M. ruber* CGMCC 10910. The results showed that the yield of yellow pigments (both extracellular and intracellular) and cell growth under oxidative conditions were significantly increased (Fig. 3b–d), and it has been reported that *Monascus* pigment production was coupled with cell growth [47]; thus, the HIO condition increased yellow pigment production primarily by enhancing cell growth. In contrast, the reducing environment inhibited the biosynthesis of yellow pigments (Fig. 3), and the yield of intracellular yellow pigments under reducing conditions was decreased in the later phase of fermentation, which resulted in a lower yield. Based on the molecular structure, it is possible that yellow pigments were formed by hydrogenation of orange pigments [43]. Under reducing conditions, the decrease of yellow pigments in the later phase of fermentation could be explained by their transformation into orange pigments. However, the glucose consumption under reducing conditions was enhanced (Fig. 3b), which indicated that there was less flux through the biosynthetic pathway of yellow pigments, and that useless byproducts such as alcohol and lactate were generated [26]. These results indicated that extracellular ORP could influence metabolic flux, which is consistent with reported studies [24, 28, 48].

The intracellular ORP, generally represented by the ratio of NADH/NAD+, has been reported to be influenced by varying the extracellular ORP [24]. In this study, the ratio of NADH/NAD+ in *M. ruber* CGMCC 10910 was significantly influenced by H_2_O_2_ addition (Fig. 4). During the fermentation process, H_2_O_2_ either accepted electrons from NADH directly, or was degraded to H_2_O and O_2_. As a result, a portion of NADH was oxidized to NAD+ by H_2_O_2_ and it achieved a redox balance so that many metabolites, such as NADH dehydrogenases and inefficient terminal oxidases (cytochrome *bd*), were not needed to balance NADH/NAD+ metabolism under HIO conditions [49]. It was speculated that H_2_O_2_ regulating NADH/NAD+ ratios maybe one of the reasons why DCW and yellow pigment yield were increased under HIO conditions (Fig. 3). During the submerged fermentation of *Monascus purpureus*, high stirring speed damages the mycelium and results in a lower pigments yield [50]. And the mycelium morphology of *Monascus anka* plays an important role in polyketides production [51]. Our study found that electron acceptors can be provided by H_2_O_2_ and without increasing stirring speed, which would damage the mycelium morphology of *M. ruber* CGMCC 10910. Moreover, it was reported that yellow pigments are the reduction product of orange pigments [43], in which two of the C–C double bonds in the conjugated chain of two orange pigments are reduced to C–C single bonds and reducing power (NADH or NADPH) is required in this reduction reaction [12]. Thus, the ratio of NADH/NAD+ under oxidative conditions was lower than that of the control (Fig. 4).

The biosynthesis of *Monascus* pigment requires many primary metabolites, such as acetyl-CoA, malonyl-CoA, NADH, and NADPH [52]. NADPH acted as reducing power used for polyketide and lipid biosynthesis [31], which was mainly produced by PPP, which is a branch pathway of glycometabolism [29]. The activities of key enzymes involved in the glycolysis pathway and PPP were analysed in this study. The activity of PFK under HIO conditions was lower than that of the control group (Fig. 6a), which indicates that PFK was allosterically inhibited by higher metabolite concentrations in the glycolysis pathway under HIO conditions [53]. G6PDH activity under oxidative conditions was higher than that of the control group (Fig. 6b), which indicated that the metabolites involved in PPP under oxidative condition were significantly up-regulated. These results showed that *M. ruber* CGMCC 10910 redirects its metabolic flux from the glycolysis pathway to the PPP, and more energy (NADPH) could be produced for polyketide and lipid biosynthesis [28, 35, 36], which is consistent with reported studies that show that metabolic flux was altered from the glycolysis pathway to the PPP, and an abundance of precursors were available for validamycin A biosynthesis in *Streptomyces hygroscopicus* 5008 fermentation in response to various oxidant treatments [38, 39]. Moreover, analysis of the relative expression levels of the pigment biosynthetic genes showed that the genes *MpFasA2*, *MpFasB2*, *MpPKS5*, *mppA*, *mppB*, *mppD*, *mppE*, and *mppR1* were significantly up-regulated under HIO conditions (*p* < 0.01 or *p* < 0.05) (Fig. 5). Thus, HIO conditions increase yellow pigment accumulation via up-regulation of the transcript levels of relative genes and improving the precursor concentrations for pigment biosynthesis.

## Conclusions

Extracellular oxidative conditions caused by H_2_O_2_ addition at suitable fermentation time points can improve the yield of extracellular water-soluble yellow pigments in submerged fermentation with *M. ruber* CGMCC 10910. Cell growth and extracellular water-soluble yellow pigment production of *M. ruber* CGMCC 10910 under HIO conditions were enhanced, while these parameters were inhibited in reducing conditions induced by dithiothreitol. Intracellular ORP (NADH/NAD+ rates) was decreased by H_2_O_2_ addition; fewer metabolites were used to balance NADH/NAD+, and more energy could thus be directed to cell growth and yellow pigment production instead of unusable metabolite production. The transcription levels of relevant pigment biosynthesis genes were up-regulated under HIO conditions; moreover, the activity of G6PDH was increased while PFK activity was inhibited. HIO conditions increased yellow pigment accumulation via up-regulation of the transcription levels of relevant genes and improving the precursor concentrations of yellow pigment biosynthesis through redirection of metabolic flux, which provides a potential improved strategy for producing water-soluble *Monascus* yellow pigments.

## Additional files

**Additional file 1: Table S1.** Primers for RT-qPCR analyzing pigments biosynthetic genes.

**Additional file 2: Figure S1.** Mass spectra and UV–visible spectra of extracellular yellow pigments detected by LC–MS and HPLC-PDA.

**Additional file 3: Figure S2.** Mass spectra and UV–visible spectra of intracellular yellow pigment monascin and ankaflavin detected by LC–MS and HPLC-PDA.

**Additional file 4: Figure S3.** HPLC-PDA chromatogram of intracellular yellow pigments and monascin standard curve detected by HPLC-PDA.

## Abbreviations

ORP: oxidoreduction potential
DTT: dithiothreitol
HIO: H_2_O_2_ induced oxidative
PPP: pentose phosphate pathway
AU: absorbance units
DCW: dry cell weight
PFK: 6-phosphofructokinase
G6PDH: glucose-6-phosphate dehydrogenase

## References

[1] Patakova P (2013). *Monascus* secondary metabolites: production and biological activity. J Ind Microbiol Biotechnol.

[2] Su NW, Lin YL, Lee MH, Ho CY (2005). Ankaflavin from *Monascus*-fermented red rice exhibits selective cytotoxic effect and induces cell death on Hep G2 cells. J Agric Food Chem.

[3] Hsu YW, Hsu LC, Liang YH, Kuo YH, Pan TM (2010). Monaphilones A–C, three new antiproliferative azaphilone derivatives from *Monascus purpureus* NTU 568. J Agric Food Chem.

[4] Hsu L, Liang Y, Hsu Y, Kuo Y, Pan T (2013). Anti-inflammatory properties of yellow and orange pigments from *Monascus* purpureus NTU568. J Agric Food Chem.

[5] Lee C, Wen J, Hsu Y, Pan T (2013). *Monascus*-fermented yellow pigments monascin and ankaflavin showed antiobesity effect via the suppression of differentiation and lipogenesis in obese rats fed a high-fat diet. J Agric Food Chem.

[6] Shi Y, Liao VH, Pan T (2012). Monascin from red mold dioscorea as a novel antidiabetic and antioxidative stress agent in rats and *Caenorhabditis elegans*. Free Radical Biol Med.

[7] Lee C, Lin P, Hsu Y, Pan T (2015). *Monascus*-fermented monascin and ankaflavin improve the memory and learning ability in amyloid β-protein intracerebroventricular-infused rat via the suppression of Alzheimer’s disease risk factors. J Funct Foods.

[8] Hsu WH, Pan TM (2014). Treatment of metabolic syndrome with ankaflavin, a secondary metabolite isolated from the edible fungus *Monascus* spp. Appl Microbiol Biotechnol.

[9] Cheng CF, Pan TM (2016). Ankaflavin and monascin induce apoptosis in activated hepatic stellate cells through suppression of the Akt/NF-kappaB/p38 signaling pathway. J Agric Food Chem.

[10] Chen G, Wu Z (2016). Production and biological activities of yellow pigments from *Monascus* fungi. World J Microbiol Biotechnol.

[11] Qingqing Z, Di Z, Wenjing T, Chao Y (2015). Photostability of water-soluble and alcohol-soluble *Monascus* Pigments. Food Sci.

[12] Edward J, Paul R, Elaine F. Reduced *Monascus* pigment derivatives as yellow food colorats. United States patent US 5,013,564. 1991.

[13] Barbosa RN, Leong SL, Vinnere-Pettersson O, Chen AJ, Souza-Motta CM, Frisvad JC, Samson RA, Oliveira NT, Houbraken J (2017). Phylogenetic analysis of *Monascus* and new species from honey, pollen and nests of stingless bees. Stud Mycol.

[14] Jiang Y, Li HB, Chen F, Hyde KD (2005). Production potential of water-soluble *Monascus* red pigment by a newly isolated *Penicillium* sp. J Agric Technol.

[15] Zhenqiang W, Gong C, Meihua W. Screening and application of *Monascus* strains with high yield of extracellular yellow pigment. China Patent No. CN201510449543.6

[16] Klinsupa W, Phansiri S, Thongpradis P, Yongsmith B, Pothiratana C (2016). Enhancement of yellow pigment production by intraspecificprotoplast fusion of *Monascus* spp. yellow mutant (ade−) and whitemutant (prototroph). J Biotechnol.

[17] Ruijie L, Yumei X, Siqin H, Lei W, Suo C, Mengxiang G, Li L (2016). Optimization of liquid-state fermentation conditions for the production of water soluble yellow *Monascus* pigment. China Brewing.

[18] Shi K, Song D, Chen G, Pistolozzi M, Wu Z, Quan L (2015). Controlling composition and color characteristics of *Monascus* pigments by pH and nitrogen sources in submerged fermentation. J Biosci Bioeng.

[19] Chen G, Shi K, Song D, Quan L, Wu Z (2015). The pigment characteristics and productivity shifting in high cell density culture of *Monascus anka* mycelia. BMC Biotechnol.

[20] Wang M, Huang T, Chen G, Wu Z (2017). Production of water-soluble yellow pigments via high glucose stress fermentation of *Monascus ruber* CGMCC 10910. Appl Microbiol Biotechnol.

[21] Huang T, Wang M, Shi K, Chen G, Tian X (2017). Metabolism and secretion of yellow pigment under high glucose stress with *Monascus ruber*. AMB Express.

[22] Berovic M (1999). Scale-up of citric acid fermentation by redox potential control. Biotechnol Bioeng.

[23] Du C, Zhang Y, Li Y, Cao ZA (2007). Novel redox potential-based screening strategy for rapid isolation of *Klebsiella pneumoniae* mutants with enhanced 1,3-propanediol-producing capability. Appl Environ Microb.

[24] Liu CG, Xue C, Lin YH, Bai FW (2013). Redox potential control and applications in microaerobic and anaerobic fermentations. Biotechnol Adv.

[25] Os-Rivera SJB, Bennett GN, San K (2002). The effect of increasing NADH availability on the redistribution of metabolic fluxes in *Escherichia coli* chemostat cultures. Metab Eng.

[26] Green J, Paget MP (2004). Bacterial redox sensors. Nat Rev Microbiol.

[27] González-Siso MI, García-Leiro A, Tarrío N, Cerdán ME (2009). Sugar metabolism, redox balance and oxidative stress response in the respiratory yeast *Kluyveromyces lacti*. Microb Cell Fact.

[28] Zhang X, Xue C, Zhao F, Li D, Yin J (2014). Suitable extracellular oxidoreduction potential inhibit rex regulation and effect central carbon and energy metabolism in *Saccharopolyspora spinosa*. Microb Cell Fact.

[29] Wasylenko TM, Ahn WS, Stephanopoulos G (2015). The oxidative pentose phosphate pathway is the primary source of NADPH for lipid overproduction from glucose in *Yarrowia lipolytica*. Metab Eng.

[30] Shao Y (2009). Characteristic analysis of transformants in T-DNA mutation library of *Monascus ruber*. World J Microbiol Biotechnol.

[31] Balakrishnan B, Karki S, Chiu S, Kim H, Suh J, Nam B, Yoon Y, Chen C, Kwon H (2013). Genetic localization and in vivo characterization of a *Monascus* azaphilone pigment biosynthetic gene cluster. Appl Microbiol Biotechnol.

[32] Shao Y, Lei M, Mao Z, Zhou Y, Chen F (2014). Insights into *Monascus* biology at the genetic level. Appl Microbiol Biotechnol.

[33] Balakrishnan B, Kim H, Suh J, Chen C, Liu K, Park S, Kwon H (2014). *Monascus* azaphilone pigment biosynthesis employs a dedicated fatty acid synthase for short chain fatty acyl moieties. J Korean Soc Appl Biol Chem.

[34] Liu J, Zhou Y, Yi T, Zhao M, Xie N, Lei M, Liu Q, Shao Y, Chen F (2016). Identification and role analysis of an intermediate produced by a polygenic mutant of *Monascus* pigments cluster in *Monascus ruber* M7. Appl Microbiol Biotechnol.

[35] Balakrishnan B, Park S, Kwon H (2017). A reductase gene mppE controls yellow component production in azaphilone polyketide pathway of *Monascus*. Biotechnol Lett.

[36] Balakrishnan B, Suh J, Park S, Kwon H (2014). Delineating *Monascus* azaphilone pigment biosynthesis: oxidoreductive modifications determine the ring cyclization pattern in azaphilone biosynthesis. RSC ADV.

[37] Wang L, Zhang J, Cao Z, Wang Y, Gao Q (2015). Inhibition of oxidative phosphorylation for enhancing citric acid production by *Aspergillus niger*. Microb Cell Fact.

[38] Liao Y, Wei ZH, Bai L, Deng Z, Zhong JJ (2009). Effect of fermentation temperature on validamycin A production by *Streptomyces hygroscopicus* 5008. J Biotechnol.

[39] Wei Z, Bai L, Deng Z, Zhong J (2011). Enhanced production of validamycin A by H_2_O_2_-induced reactive oxygen speciesin fermentation of *Streptomyces hygroscopicus* 5008. Bioresour Technol.

[40] Teng SS, Feldheim W (1998). Analysis of anka pigments by liquid chromatography with diode array detection and tandem mass spectrometry. Chromatographia.

[41] Zheng Y, Xin Y, Guo Y (2009). Study on the fingerprint profile of *Monascus* products with HPLC–FD, PAD and MS. Food Chem.

[42] Chen W, He Y, Zhou Y, Shao Y, Feng Y, Li M, Chen F (2015). Edible filamentous fungi from the species *Monascus*: early traditional fermentations, modern molecular biology, and future genomics. Compr Rev Food Sci Food Saf.

[43] Juzlova P, Martinkova L, Kren V (1996). Secondary metabolites of the fungus *Monascus*: a review. J Ind Microbiol.

[44] Shao Y, Li Q, Zhou Y, Chen F (2017). Effects of an alternative oxidase gene on conidia viability under external stresses in *Monascus ruber* M7. J Basic Microbiol.

[45] Qian ZG, Zhao ZJ, Tian WH, Xu Y, Zhong JJ (2004). Novel synthetic jasmonates as highly efficient elicitors for taxoid production by suspension cultures of *Taxus chinensis*. Biotechnol Bioeng.

[46] Babu BK, Atiyeh HK, Wilkins MR, Huhnke RL (2010). Effect of the reducing agent dithiothreitol on ethanol and acetic acid production by *Clostridium* strain P11 using simulated biomass-based syngas. Biol Eng.

[47] Vendruscolo F, Schmidell W, De OD, Ninow JL (2016). Kinetic of orange pigment production from *Monascus ruber* on submerged fermentation. Bioprocess Biosyst Eng.

[48] Riondet C, Cachon R, Waché Y, Alcaraz G, Diviès C (2000). Extracellular oxidoreduction potential modifies carbon and electron flow in *Escherichia coli*. J Bacteriol.

[49] Brekasis D, Paget MS (2003). A novel sensor of NADH/NAD+ redox poise in *Streptomyces coelicolor* A3. EMBO J.

[50] Jun L, Bobo Z, Gangrong X, Peter CC (2017). Enhanced production of natural yellow pigments from *Monascus* by liquid culture: a study of the fermentation conditions and mycelial morphology. J Biosci Bioeng.

[51] Chen G, Huang T, Bei Q, Tian X, Wu Z (2017). Correlation of pigment production with mycelium morphology in extractive fermentation of *Monascus anka* GIM 3.592. Process Biochem.

[52] Ruiz B, Chávez A, Forero A, García-Huante Y, Romero A, Sánchez M, Rocha D, Sánchez B, Rodríguez-Sanoja R, Sánchez S, Langley E (2010). Regulationby carbon source. Crit Rev Microbiol.

[53] Peng L, Shimizu K (2003). Global metabolic regulation analysis for *Escherichia coli* K12 based on protein expression by 2-dimensional electrophoresis and enzyme activity measurement. Appl Microbiol Biotechnol.

