# Supplementary material for: Changing oxidoreduction potential to improve water-soluble yellow pigment production with Monascus ruber CGMCC 10910
Source: Microb Cell Fact. 2017 Nov 21;16:208. doi: 10.1186/s12934-017-0828-0 (PMC5697053; doi:10.1186/s12934-017-0828-0)
Supplement: Supplementary file 1 — Additional file 1: Table S1. Primers for RT-qPCR analyzing pigments biosynthetic genes. [file 12934_2017_828_MOESM1_ESM.doc]

**Additional file 1: Table S1**

**Table S1.** Primers for RT-qPCR analyzing pigments biosynthetic genes

| Primers name | Primers sequences (5′→3′) |
| --- | --- |
| *actin* F | TTCGAGACCTTCAACGCCC |
| *actin* R | ACCCTCGTAGATGGGAACGA |
| *MpPKS5*F | TGTCCGACGAGTTTCTGCAA |
| *MPPKS5*R | TATCAACGCTGCTTGGGCAT |
| *mppR1*F | TCTGCAGTATGCCATGTGGG |
| *mppR1*R | ATGGCACCGTCACTTAGCTC |
| *mppA F* | GCTGATTTCGGGTGTTTC |
| *mppA R* | GCTTGTTACTTTTGCTGTTC |
| *mppB* F | CGTCTCGCCCGATAACTTCA |
| *mppB* R | TTGACAGACGGGTCGAAGTC |
| *mppC* F | CAGTCCTCGTCCCTTCCAGT |
| *mppC* R | CCACGGTGAAGGATGTCGAG |
| *mppD* F | TCAACACGGGAGATGCTGTC |
| *mppD* R | GCCAAAGGACAGGAGCAGAT |
| *mppE* F | CTTCCCGATGCCGTTGTGAT |
| *mppE* R | CGTCTCGTGGATCATCTCGT |
| *mppR2*F | ACGAAACCCTCCATGACACC |
| *mppR2*R | TGCAGACAGCCTTGTGGTAG |
| *MpFasA2* F | ATGGATCGCCCGATCTTGTC |
| *MpFasA2* R | CTTTGTCGAGTCCGCTGGAT |
| *MpFasB2* F | CCTCCAGGGATTACAACCCG |
| *MpFasB2* R | ATTCAATGCCAGGTGCTCCA |
