# Supplementary material for: Changing oxidoreduction potential to improve water-soluble yellow pigment production with Monascus ruber CGMCC 10910
Source: Microb Cell Fact. 2017 Nov 21;16:208. doi: 10.1186/s12934-017-0828-0 (PMC5697053; doi:10.1186/s12934-017-0828-0)
Supplement: Supplementary file 2 — Additional file 2: Figure S1. Mass spectra and UV–visible spectra of extracellular yellow pigments detected by LC–MS and HPLC-PDA. [file 12934_2017_828_MOESM2_ESM.doc]

**Additional file 2: Figure S1**

**Fig. S1** Mass spectra and UV-Visible spectra of extracellular yellow pigments detected by LC-MS and HPLC-PDA.
