# Supplementary material for: Changing oxidoreduction potential to improve water-soluble yellow pigment production with Monascus ruber CGMCC 10910
Source: Microb Cell Fact. 2017 Nov 21;16:208. doi: 10.1186/s12934-017-0828-0 (PMC5697053; doi:10.1186/s12934-017-0828-0)
Supplement: Supplementary file 4 — Additional file 4: Figure S3. HPLC-PDA chromatogram of intracellular yellow pigments and monascin standard curve detected by HPLC-PDA. [file 12934_2017_828_MOESM4_ESM.doc]

**Additional file 4: Figure S3**

**Fig.** **S3** HPLC-PDA chromatogram of intracellular yellow pigments and Monascin standard curve detected by HPLC-PDA. 1, monascin. 2, ankaflavin. 3, rubropunctation. 4, monascorubrin.
